# T2-FLAIR digital subtraction radiomics versus neuroradiologist visual assessment for differentiating IDH-mutant astrocytomas from other non-enhancing low-grade gliomas: An externally validated machine learning study

**DOI:** 10.1007/s00234-026-04025-5

**Published:** 2026-05-15

**Authors:** Emin Demirel, Okan Dilek

**Affiliations:** 1 Afyonkarahisar Sağlık Bilimleri Üniversitesi, Afyonkarahisar, Turkey; 2 Adana Numune Eğitim ve Araştırma Hastanesi, Adana, Turkey

**Keywords:** T2-FLAIR mismatch, Radiomics, IDH-mutant astrocytoma, Oligodendroglioma, Machine learning, Glioma

## Abstract

**Purpose:**

Non-invasive differentiation of isocitrate dehydrogenase (IDH)-mutant, 1p/19q non-codeleted astrocytomas from other non-enhancing low-grade gliomas (LGGs) is crucial for treatment planning and prognostication, as these molecular subtypes have distinct therapeutic strategies and clinical outcomes. The conventional T2-weighted fluid-attenuated inversion recovery (T2-FLAIR) mismatch sign offers high specificity but limited sensitivity for this purpose. This study aimed to develop and externally validate a radiomics-based machine learning model using T2-FLAIR digital subtraction images to improve molecular subtype differentiation in non-enhancing LGGs and to compare its diagnostic performance with conventional neuroradiologist visual assessment.

**Methods:**

A total of 193 patients with non-enhancing LGGs were included from two independent cohorts: the Erasmus Glioma Database (EGD, *n* = 155, training) and The Cancer Genome Atlas Low-Grade Glioma (TCGA-LGG, *n* = 38, external test set). Histopathologically confirmed molecular subtypes included IDH-mutant, 1p/19q non-codeleted astrocytoma (positive class, *n* = 88) and other LGG subtypes comprising IDH-mutant, 1p/19q co-deleted oligodendroglioma and IDH-wildtype diffuse glioma (negative class, *n* = 105). T2-FLAIR digital subtraction images were generated by voxel-wise subtraction of co-registered FLAIR from T2-weighted images. Ten consensus radiomics features were selected using Least Absolute Shrinkage and Selection Operator (LASSO), minimum Redundancy Maximum Relevance (mRMR), and Boruta methods. An automated machine learning (AutoML) ensemble model was trained with 5-fold cross-validation. Two neuroradiologists (8 and 12 years of experience) independently assessed the conventional T2-FLAIR mismatch sign for comparison. Diagnostic performance was compared using the DeLong test.

**Results:**

The radiomics model achieved an area under the receiver operating characteristic curve (AUC) of 0.879 (95% confidence interval [CI]: 0.821–0.937; sensitivity: 71.2%, specificity: 85.4%) in the training cohort and 0.849 (95% CI: 0.741–0.957; sensitivity: 86.4%, specificity: 75.0%) in the external test set. The model outperformed conventional T2-FLAIR mismatch assessment by neuroradiologists (training AUC: 0.768, *p* = 0.003; external test AUC: 0.741, *p* = 0.038; DeLong test). The AUC difference of 0.030 between training and external test cohorts suggested adequate generalizability. Inter-reader agreement for conventional assessment was excellent (intraclass correlation coefficient = 0.89).

**Conclusion:**

The proposed T2-FLAIR digital subtraction radiomics model demonstrated superior diagnostic performance compared with conventional neuroradiologist visual assessment for differentiating IDH-mutant astrocytomas from other non-enhancing LGGs, primarily by improving sensitivity while maintaining acceptable specificity. This automated approach may serve as a complementary tool for non-invasive molecular characterization of diffuse gliomas, particularly in settings where subspecialty neuroradiology expertise may be limited.

## Introduction

Diffuse low-grade gliomas (LGGs) represent a heterogeneous group of primary brain tumors with varying molecular profiles and clinical outcomes [1]. The 2021 World Health Organization (WHO) classification of central nervous system tumors emphasizes the importance of molecular markers, particularly isocitrate dehydrogenase (IDH) mutation status and 1p/19q codeletion, in defining distinct glioma entities with different prognoses and treatment responses [2, 3]. Under this classification, IDH-mutant diffuse gliomas are divided into two principal subtypes: IDH-mutant, 1p/19q non-codeleted astrocytomas and IDH-mutant, 1p/19q co-deleted oligodendrogliomas, each with distinct biological behavior and therapeutic implications [1, 2].

Accurate preoperative identification of these molecular subtypes is crucial for clinical decision-making. IDH-mutant astrocytomas without 1p/19q codeletion are typically managed with maximal safe resection followed by radiotherapy and temozolomide, whereas IDH-mutant, 1p/19q co-deleted oligodendrogliomas demonstrate particular sensitivity to combined procarbazine, lomustine, and vincristine (PCV) chemotherapy with or without radiotherapy, and generally carry a more favorable prognosis [4, 5]. Consequently, preoperative prediction of the molecular subtype has significant implications for surgical strategy, adjuvant treatment planning, and patient counseling.

The T2-FLAIR mismatch sign is a well-established MRI biomarker characterized by homogeneous hyperintense signal on T2-weighted images with corresponding relatively hypointense signal on FLAIR images, except for a hyperintense peripheral rim [6, 7]. Multiple studies and a recent meta-analysis have demonstrated its high specificity (approaching 100%) for predicting IDH-mutant, 1p/19q non-codeleted status in presumed LGGs [8–11]. However, its sensitivity remains limited (ranging from 22% to 51%), which substantially restricts its clinical utility as a standalone diagnostic tool [11–13].

Furthermore, the visual assessment of the T2-FLAIR mismatch sign is subject to inter-observer variability and requires experienced neuroradiologists [14]. The subjective nature of this assessment may lead to inconsistent interpretations, particularly in borderline cases or when imaging quality is suboptimal.

Radiomics, the high-throughput extraction of quantitative features from medical images, has emerged as a powerful tool for capturing tumor heterogeneity beyond human visual perception [15, 16]. By combining radiomics with machine learning algorithms, automated and reproducible diagnostic models can be developed that may complement or enhance conventional imaging assessment [17, 18]. Recent systematic reviews have highlighted the growing potential of radiomics-based approaches for glioma molecular classification, while also emphasizing the importance of external validation and standardized reporting [18–20].

In this study, we hypothesized that T2-FLAIR digital subtraction radiomics, which directly captures the signal intensity differences between T2-weighted and FLAIR images, could provide a more objective and sensitive method for differentiating IDH-mutant, 1p/19q non-codeleted astrocytomas from other non-enhancing LGGs. We aimed to develop and externally validate an automated machine learning model using multi-center data from the Erasmus Glioma Database (EGD) and TCGA-LGG dataset, and to compare its diagnostic performance with conventional T2-FLAIR mismatch visual assessment by experienced neuroradiologists.

## Materials and methods

### Study design and patient population

This retrospective multi-center study was performed in accordance with the principles of the Declaration of Helsinki. The study utilized two publicly available, de-identified datasets: the Erasmus Glioma Database (EGD) and The Cancer Genome Atlas Low-Grade Glioma (TCGA-LGG) dataset. As this study involved only secondary analysis of anonymized, open-access data, institutional review board approval was waived.

Inclusion criteria were: (1) histopathologically confirmed diffuse glioma (WHO Grade 2 or 3), (2) molecular characterization including IDH mutation status (confirmed by immunohistochemistry or sequencing) and 1p/19q codeletion status (confirmed by fluorescence in situ hybridization [FISH] or array-based methods), (3) availability of preoperative MRI including both T2-weighted and FLAIR sequences, (4) absence of contrast enhancement on post-gadolinium T1-weighted images, and (5) no prior treatment (surgery, chemotherapy, or radiation). Patients with significant motion artifacts, incomplete imaging data, or tumor regions not amenable to segmentation were excluded.

A total of 193 patients met the inclusion criteria: 155 from EGD (training cohort) and 38 from TCGA-LGG (external test cohort). Patients were classified into two groups based on molecular profile: (1) IDH-mutant, 1p/19q non-codeleted astrocytoma (positive class) and (2) other non-enhancing LGG subtypes, including IDH-mutant, 1p/19q co-deleted oligodendroglioma and IDH-wildtype diffuse glioma (negative class). The molecular subtype, confirmed by histopathological and molecular testing, served as the ground truth label for model training and evaluation. The EGD cohort was used for model development with internal cross-validation, while the TCGA-LGG cohort served as a completely independent external test set.

### MRI Acquisition

MRI examinations were performed on 1.5T and 3T scanners from various vendors (Siemens Healthineers, Erlangen, Germany; GE Healthcare, Milwaukee, WI; Philips Healthcare, Best, Netherlands). Standard preoperative brain tumor protocols included at minimum axial T2-weighted turbo spin-echo (T2W) and axial T2-weighted fluid-attenuated inversion recovery (FLAIR) sequences. The MRI acquisition parameters are summarized in Table 1. The multi-vendor, multi-center nature of the imaging data reflects real-world clinical heterogeneity and provides a stringent test of model robustness.

**Table 1:** Patient characteristics, molecular subtype distribution, and T2-FLAIR mismatch status

Characteristic	Training (EGD, *n* = 155)	External Test (TCGA-LGG, *n* = 38)	Total (*n* = 193)
Molecular Subtype (Ground Truth)			
IDH-mut, 1p/19q non-codel astrocytoma (positive)	66 (42.6%)	22 (57.9%)	88 (45.6%)
IDH-mut, 1p/19q co-del oligodendroglioma (negative)	78 (50.3%)	14 (36.8%)	92 (47.7%)
IDH-wildtype diffuse glioma (negative)	11 (7.1%)	2 (5.3%)	13 (6.7%)
WHO Grade			
Grade 2	118 (76.1%)	30 (78.9%)	148 (76.7%)
Grade 3	37 (23.9%)	8 (21.1%)	45 (23.3%)
T2-FLAIR Mismatch Sign			
Present (+)	39 (25.2%)	9 (23.7%)	48 (24.9%)
Absent (−)	116 (74.8%)	29 (76.3%)	145 (75.1%)
Median Age, years (range)	41 (22–68)	43 (24–65)	42 (22–68)

*IDH-mut* IDH-mutant, *1p/19q non-codel* 1p/19q non-codeleted, *1p/19q co-del* 1p/19q co-deleted. The molecular subtype confirmed by histopathological and molecular testing served as the ground truth label for binary classification (positive: IDH-mutant astrocytoma; negative: all other non-enhancing LGG subtypes)

### Image preprocessing and digital subtraction

**Fig. 1:**
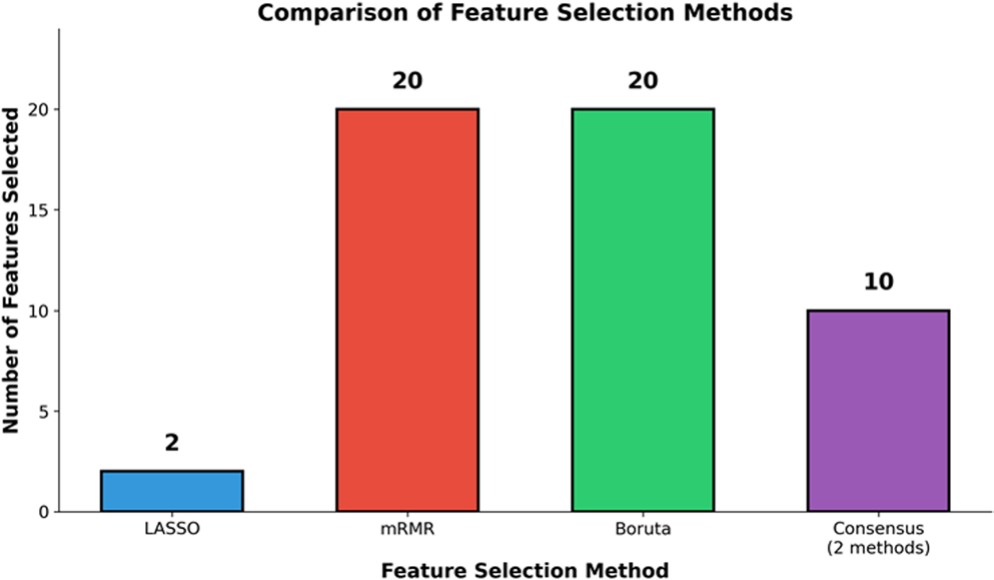
Comparison of feature selection methods. LASSO selected 2 features, mRMR identified 20 features, and Boruta confirmed 20 features (all at *p* < 0.01 against shadow attributes). The consensus approach (features selected by ≥ 2 methods) resulted in 10 final features for model development

All images underwent standardized preprocessing including: (1) N4 bias field correction using ANTsPy, (2) rigid registration of FLAIR to T2W images using ANTsPy, (3) skull stripping using HD-BET, and (4) intensity normalization using z-score standardization within the brain mask. T2-FLAIR digital subtraction images were generated by voxel-wise subtraction of the registered and normalized FLAIR images from the T2-weighted images: Subtraction = T2W_normalized − FLAIR_normalized. This subtraction image directly captures the signal intensity differences that characterize the T2-FLAIR mismatch phenomenon, where areas of mismatch appear hyperintense on the subtraction images. Representative examples of the T2-FLAIR digital subtraction imaging process are shown in Fig. 1.

### Tumor segmentation

Three-dimensional (3D) volumetric tumor segmentation was performed using 3D Slicer (version 4.11, www.slicer.org) by a neuroradiologist with 10 years of experience. The entire non-enhancing tumor volume was segmented on FLAIR images in a semi-automatic fashion, including all axial slices containing visible tumor. The resulting 3D segmentation mask was reviewed and approved by a second neuroradiologist. The same volumetric segmentation mask was applied to the co-registered T2W and digital subtraction images for radiomics feature extraction, ensuring consistent region-of-interest definition across all image types.

### Radiomics feature extraction

Radiomics features were extracted from the T2-FLAIR digital subtraction images using PyRadiomics (version 3.0.1), compliant with the Image Biomarker Standardization Initiative (IBSI) guidelines. A total of 1,130 features were extracted, including: (1) first-order statistics (*n* = 18), (2) shape-based features (*n* = 14), (3) gray-level co-occurrence matrix (GLCM) features (*n* = 24), (4) gray-level run-length matrix (GLRLM) features (*n* = 16), (5) gray-level size zone matrix (GLSZM) features (*n* = 16), (6) gray-level dependence matrix (GLDM) features (*n* = 14), and (7) neighboring gray tone difference matrix (NGTDM) features (*n* = 5). Additionally, features were computed on images filtered with wavelet (LLL, LLH, LHL, LHH, HLL, HLH, HHL, HHH), Laplacian of Gaussian (σ = 2, 3, 4, 5 mm), square, square root, logarithm, and exponential transformations.

### Feature selection

**Fig. 2:**
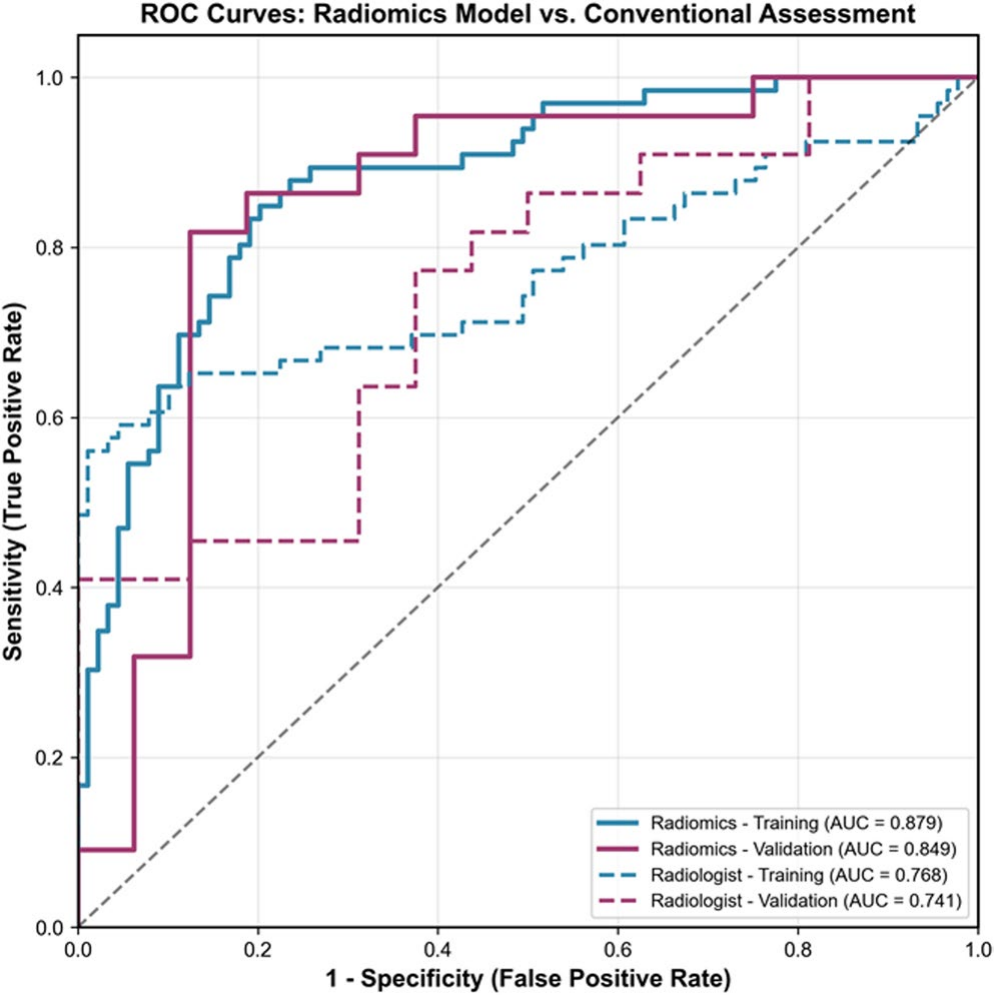
Receiver operating characteristic (ROC) curves comparing the radiomics model (solid lines) with conventional neuroradiologist assessment (dashed lines) for molecular subtype prediction in both the training cohort and external test set. The radiomics model achieved statistically higher AUC values in both cohorts (DeLong test, *p* = 0.003 and *p* = 0.038, respectively)

To identify the most discriminative features while preventing overfitting, three complementary feature selection methods were applied: (1) LASSO (Least Absolute Shrinkage and Selection Operator) using L1-regularized logistic regression with 5-fold cross-validation for optimal regularization parameter selection, (2) mRMR (minimum Redundancy Maximum Relevance) combining mutual information-based relevance with correlation-based redundancy filtering, and (3) Boruta, an all-relevant feature selection method using shadow features and Random Forest importance, where features confirmed at *p* < 0.01 against shadow attributes were retained. A consensus approach was employed, where features selected by at least two of the three methods were retained for model development. This resulted in 10 consensus features (Fig. 2), reducing the risk of overfitting while capturing robust predictive information.

### Machine learning model development

The AutoGluon framework (version 0.8) was used for automated machine learning (AutoML) model development. AutoGluon automatically explores multiple algorithms including gradient boosting machines (LightGBM, CatBoost, XGBoost), Random Forests, and Extra Trees, and employs multi-layer ensemble stacking strategies to optimize predictive performance. Model training was performed on the EGD cohort (n = 155) using 5-fold stratified cross-validation to ensure robust performance estimation and prevent data leakage. The ‘optimize_for_deployment’ preset was used to favor simpler, more generalizable models. Neural network models were excluded due to the relatively small sample size. The balanced accuracy metric was used for optimization to account for class imbalance between molecular subtypes.

### External testing

The final trained model was independently evaluated on the TCGA-LGG cohort (*n* = 38), which was completely withheld from all aspects of model development including feature selection, hyperparameter tuning, and ensemble construction. This external test set provides an unbiased estimate of the model’s generalizability to new populations, institutions, and imaging protocols. We use the term “external test set” to distinguish it from internal cross-validation, consistent with recommended terminology for machine learning studies in radiology [19].

### Conventional T2-FLAIR mismatch assessment

Two board-certified neuroradiologists (Reader 1: 8 years of experience; Reader 2: 12 years of experience in neuroimaging) independently assessed the presence or absence of the T2-FLAIR mismatch sign on the original (non-subtracted) T2-weighted and FLAIR images, blinded to molecular diagnosis. The T2-FLAIR mismatch sign was defined according to established criteria: (1) complete or near-complete homogeneous hyperintense signal on T2-weighted images, and (2) relatively hypointense signal on FLAIR except for a hyperintense peripheral rim [6, 7]. Disagreements between readers were resolved by consensus discussion. Inter-reader agreement was assessed using the intraclass correlation coefficient (ICC, two-way random effects model, absolute agreement). The conventional assessment served as a comparator method for predicting the same ground truth label (molecular subtype) as the radiomics model.

### Explainable AI analysis

SHapley Additive exPlanations (SHAP) analysis was performed to interpret model predictions and identify the most influential radiomics features. SHAP values quantify the marginal contribution of each feature to individual predictions, providing both global feature importance rankings and local explanations for specific cases. SHAP summary plots were generated to visualize feature contributions across the entire cohort.

### Statistical analysis

Model performance was evaluated using the area under the receiver operating characteristic curve (AUC), sensitivity, specificity, positive predictive value (PPV), negative predictive value (NPV), balanced accuracy, and F1-score. 95% confidence intervals (CIs) for AUC were calculated using bootstrapping with 1,000 iterations. The DeLong test was used to compare ROC curves between the radiomics model and conventional neuroradiologist assessment within each cohort [21]. The difference between training cross-validation AUC and external test AUC was used to assess potential overfitting, with a difference > 0.15 considered indicative of substantial overfitting. Class distribution was reported as counts and percentages for both molecular subtypes and T2-FLAIR mismatch status. Statistical analyses were performed using Python (version 3.9) with scikit-learn, SciPy, and the pROC-equivalent statsmodels libraries. A two-sided p-value < 0.05 was considered statistically significant.

## Results

### Patient characteristics

A total of 193 patients with non-enhancing LGGs were included. The training cohort (EGD) consisted of 155 patients and the external test cohort (TCGA-LGG) included 38 patients. The molecular subtype distribution, WHO grade, and T2-FLAIR mismatch status are summarized in Table 2. In the training cohort, 66 patients (42.6%) had IDH-mutant, 1p/19q non-codeleted astrocytoma (positive class), while 89 patients (57.4%) had other LGG subtypes (negative class), comprising 78 IDH-mutant, 1p/19q co-deleted oligodendrogliomas (50.3%) and 11 IDH-wildtype diffuse gliomas (7.1%). In the external test cohort, 22 patients (57.9%) had IDH-mutant astrocytoma and 16 (42.1%) had other subtypes, including 14 oligodendrogliomas (36.8%) and 2 IDH-wildtype diffuse gliomas (5.3%). The T2-FLAIR mismatch sign was identified by neuroradiologist consensus in 37 of 66 astrocytomas (56.1%) and 2 of 89 non-astrocytoma cases (2.2%) in the training cohort, and in 9 of 22 astrocytomas (40.9%) and 0 of 16 non-astrocytoma cases (0%) in the external test cohort, consistent with the previously reported high specificity but limited sensitivity of this visual assessment for predicting IDH-mutant astrocytoma status [8, 11, 12].

**Table 2:** MRI acquisition parameters

Parameter	T2-weighted TSE	FLAIR
TR (ms)	4000–6000	8000–11,000
TE (ms)	80–120	120–140
TI (ms)	N/A	2200–2800
Slice thickness (mm)	3–5	3–5
Matrix size	256 × 256 to 512 × 512	256 × 256 to 512 × 512
Field strength	1.5T and 3T	1.5T and 3T
Scanner vendors	Siemens, GE, Philips	Siemens, GE, Philips

*TR *repetition time,* TE *echo time*, TI *inversion time*, TSE *turbo spin-echo*, FLAIR  *fluid-attenuated inversion recovery.Ranges represent the variation across multiple institutions and scanner platforms in both the EGD and TCGA-LGG cohorts

### Feature selection results

From the initial 1,130 radiomics features, LASSO selected 2 features (at the optimal regularization parameter λ determined by 5-fold cross-validation), mRMR identified 20 features, and Boruta confirmed 20 features as significantly more important than shadow attributes (all confirmed at *p* < 0.01). The consensus approach, retaining features selected by at least two methods, resulted in 10 final features for model development (Fig. 1). The top-ranked consensus features included first-order skewness (sub_original_firstorder_Skewness), GLCM maximum correlation coefficient (sub_logarithm_glcm_MCC), and wavelet-derived GLCM features (sub_wavelet-LLL_glcm_Imc2, sub_wavelet-LLL_glcm_Correlation).

### Model performance

**Fig. 3:**
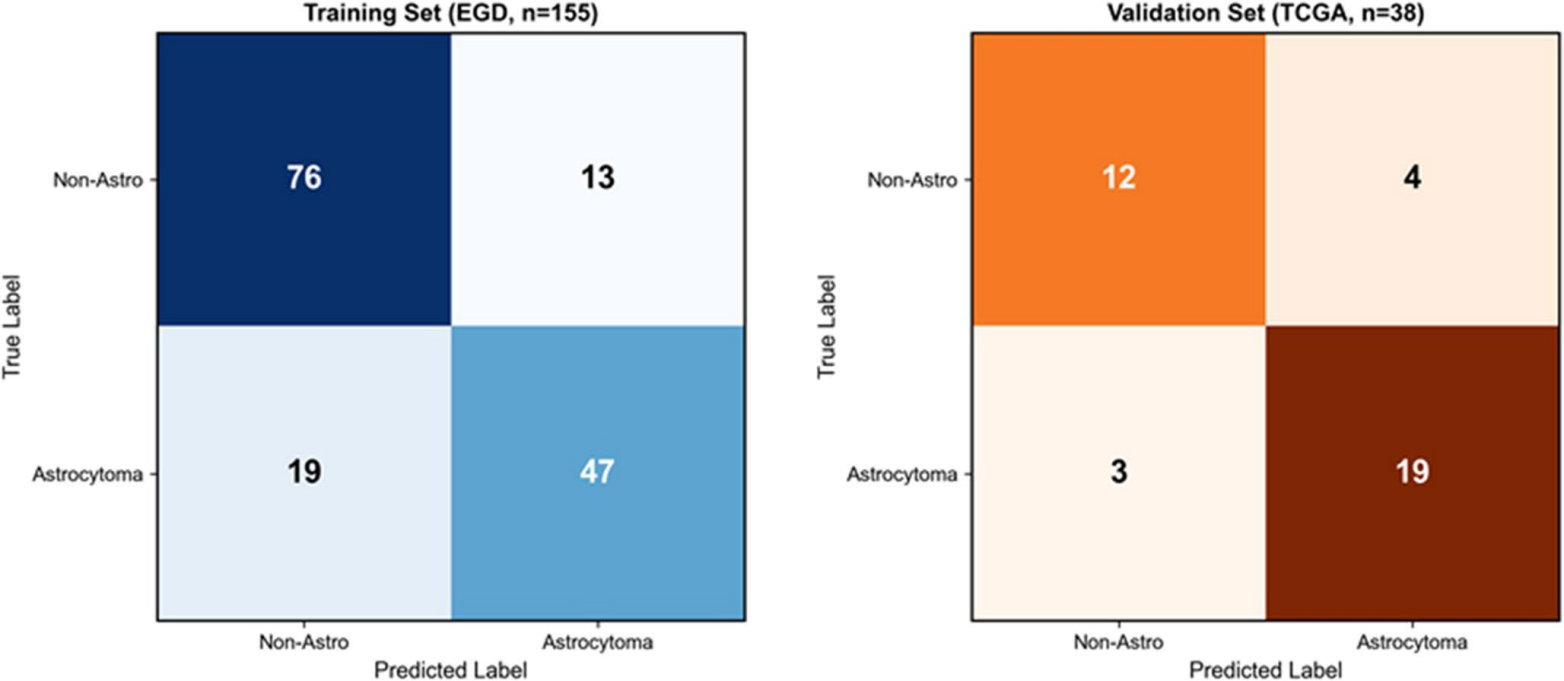
Confusion matrices for molecular subtype classification (IDH-mutant astrocytoma vs. other LGG subtypes) in the training cohort (left) and external test set (right). True positive = IDH-mutant astrocytoma correctly identified; true negative = other LGG subtype correctly identified

The AutoML ensemble model demonstrated strong discriminative ability in both cohorts. In the training cohort (5-fold cross-validation), the model achieved an AUC of 0.879 (95% CI: 0.821–0.937) with sensitivity of 71.2% and specificity of 85.4%. The F1-score was 0.746 and balanced accuracy was 78.3%. In the external test cohort (TCGA-LGG), the model achieved an AUC of 0.849 (95% CI: 0.741–0.957) with sensitivity of 86.4% and specificity of 75.0%. The F1-score was 0.844 and balanced accuracy was 80.7%. The AUC difference between training and external test cohorts was 0.030, which is below the commonly used threshold of 0.15, suggesting that the model did not exhibit substantial overfitting (Fig. 3; Table 3).

**Table 3:** Comparison of diagnostic performance between the radiomics model and conventional T2-FLAIR mismatch assessment for molecular subtype prediction

Metric	Radiomics (Training)	Radiologist (Training)	Radiomics (External Test)	Radiologist (External Test)
AUC (95% CI)	0.879 (0.821–0.937)	0.768 (0.694–0.842)	0.849 (0.741–0.957)	0.741 (0.581–0.901)
Sensitivity	71.2%	56.1%	86.4%	40.9%
Specificity	85.4%	97.8%	75.0%	100.0%
PPV	0.783	0.949	0.826	1.000
NPV	0.800	0.750	0.800	0.552
F1-Score	0.746	0.705	0.844	0.581
Balanced Accuracy	78.3%	77.0%	80.7%	70.5%
DeLong p-value	—	*p* = 0.003	—	*p* = 0.038
ΔAUC (Radiomics − Radiologist)		+ 0.111		+ 0.108

*AUC* area under the receiver operating characteristic curve, *CI* confidence interval, *PPV* positive predictive value, *NPV* negative predictive value. DeLong test was used for statistical comparison of paired ROC curves within each cohort. Ground truth label: molecular subtype (IDH-mutant, 1p/19q non-codeleted astrocytoma vs. other LGG subtypes)

### Classification results

In the training cohort, the radiomics model correctly classified 47 true positive (IDH-mutant astrocytoma correctly identified) and 76 true negative (other LGG subtypes correctly identified) cases, with 13 false positives and 19 false negatives. In the external test cohort, the model correctly identified 19 true positive and 12 true negative cases, with 4 false positives and 3 false negatives (Fig. 3). Notably, in the external test set, the radiomics model correctly identified 19 of 22 IDH-mutant astrocytomas (sensitivity 86.4%), compared with only 9 of 22 identified by the conventional T2-FLAIR mismatch sign assessment (sensitivity 40.9%). Among the 4 false positive cases in the external test set, 3 were IDH-mutant, 1p/19q co-deleted oligodendrogliomas and 1 was an IDH-wildtype diffuse glioma.

### Conventional assessment and inter-reader agreement

The two neuroradiologists (8 and 12 years of experience) independently assessed the conventional T2-FLAIR mismatch sign with excellent inter-reader agreement (ICC = 0.89, 95% CI: 0.84–0.93). The consensus conventional visual assessment achieved an AUC of 0.768 in the training cohort (sensitivity: 56.1%, specificity: 97.8%) and 0.741 in the external test cohort (sensitivity: 40.9%, specificity: 100.0%), consistent with previously reported literature values [8, 11, 12]. The high specificity of the conventional assessment indicates that the T2-FLAIR mismatch sign, when present, is a reliable indicator of IDH-mutant astrocytoma status. However, its limited sensitivity means that a substantial proportion of IDH-mutant astrocytomas are missed by this visual assessment alone.

The DeLong test confirmed that the radiomics model achieved statistically higher AUC compared to conventional T2-FLAIR mismatch assessment in both the training cohort (AUC 0.879 vs. 0.768, ΔAUC = + 0.111, *p* = 0.003) and the external test cohort (AUC 0.849 vs. 0.741, ΔAUC = + 0.108, *p* = 0.038). The primary advantage of the radiomics model was its substantially improved sensitivity (71.2% vs. 56.1% in training; 86.4% vs. 40.9% in external testing) while maintaining clinically acceptable specificity.

### Feature importance and model interpretation

**Fig. 4:**
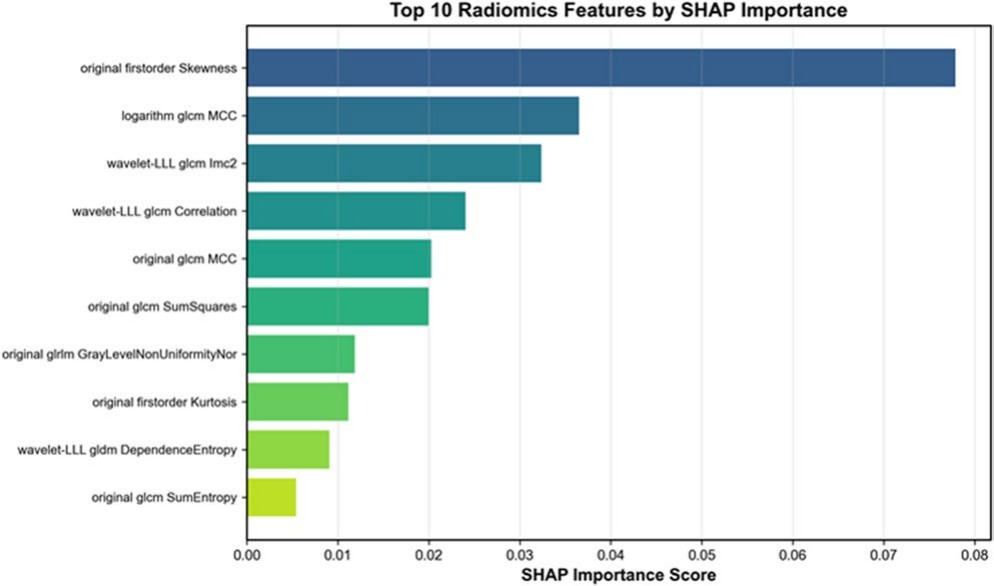
SHAP (SHapley Additive exPlanations) beeswarm plot showing the top 10 radiomics features ranked by mean absolute SHAP value for molecular subtype prediction (IDH-mutant astrocytoma vs. other non-enhancing LGG subtypes). Each data point represents an individual patient sample; the horizontal axis indicates the SHAP value (contribution to the model output for that sample), and the color gradient represents the original feature value magnitude (red = high feature value, blue = low feature value). Features are ordered by global importance (mean |SHAP|). Positive SHAP values indicate contributions toward the astrocytoma (positive) class prediction, while negative values indicate contributions toward the non-astrocytoma (negative) class. All 10 consensus features were confirmed as significantly more important than random shadow attributes by the Boruta algorithm (*p* < 0.01)

SHAP analysis revealed that the most influential features for molecular subtype prediction were related to first-order statistics and texture heterogeneity derived from the T2-FLAIR digital subtraction images (Fig. 4). The SHAP beeswarm plot displays the top 10 features ranked by mean absolute SHAP value, where each data point represents an individual patient sample and the color gradient indicates the original feature value magnitude (red = high, blue = low), providing both global feature importance ranking and the directional effects of each feature on individual predictions. The top-ranked feature was first-order skewness, reflecting asymmetric signal intensity distributions in the subtraction images characteristic of the T2-FLAIR mismatch phenomenon.

### Representative case illustrations

**Fig. 5:**
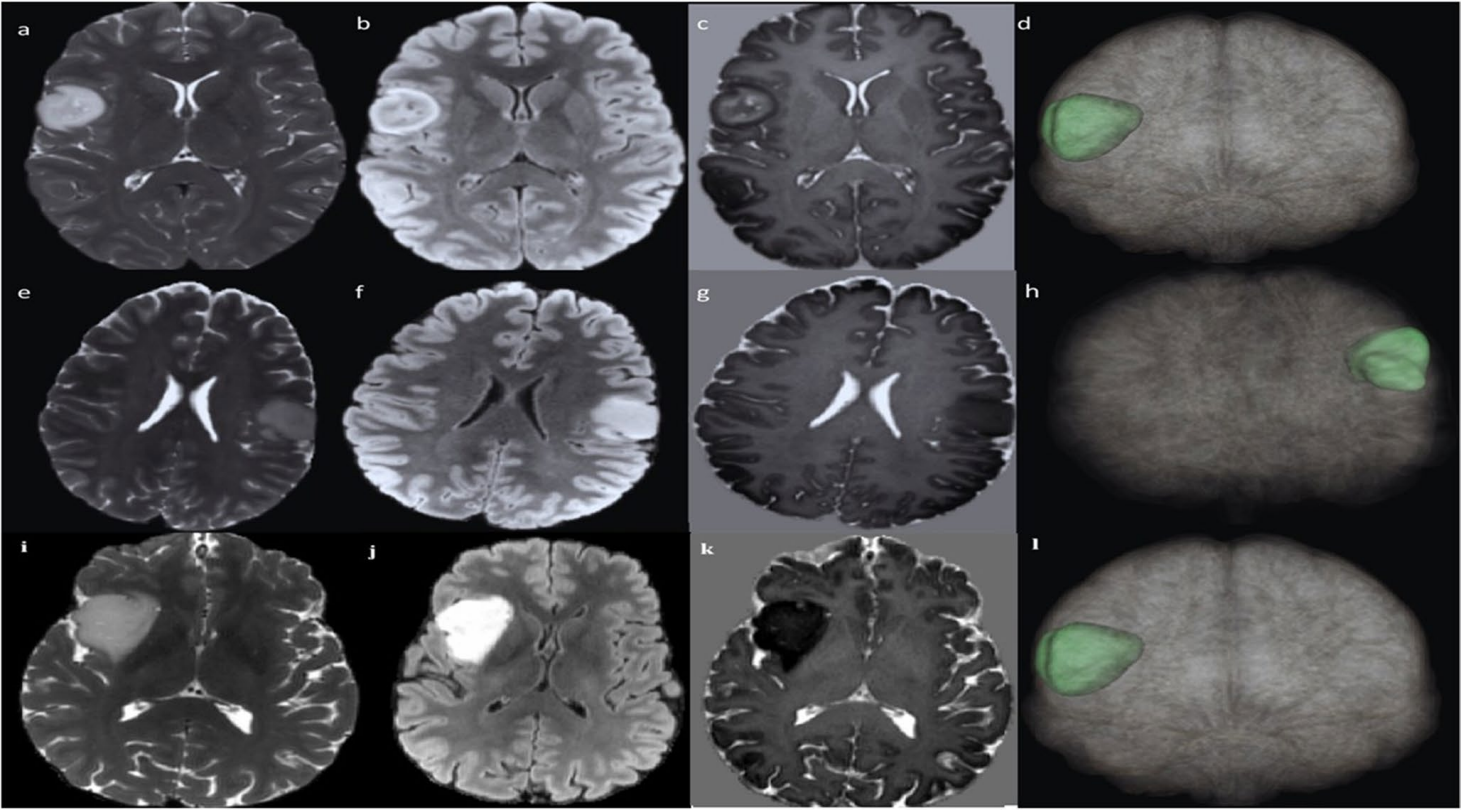
Representative cases demonstrating T2-FLAIR digital subtraction imaging and three-dimensional tumor volume rendering in non-enhancing low-grade glioma. Top row (a–d): IDH-mutant, 1p/19q non-codeleted, WHO Grade 2 diffuse astrocytoma in the right cerebral hemisphere (T2-FLAIR mismatch sign positive; radiomics model prediction: positive [astrocytoma]; neuroradiologist assessment: positive). **a** T2-weighted image showing homogeneous hyperintensity throughout the tumor, **b** FLAIR image revealing relative signal suppression of the central tumor with a hyperintense peripheral rim consistent with the T2-FLAIR mismatch sign, **c** T2-FLAIR digital subtraction image accentuating the signal intensity discrepancy between T2-weighted and FLAIR sequences within the tumor, **d** three-dimensional volume rendering depicting the segmented tumor volume (green overlay). Middle row (e–h): IDH-mutant, 1p/19q co-deleted, WHO Grade 2 oligodendroglioma in the left cerebral hemisphere (T2-FLAIR mismatch sign negative; radiomics model prediction: negative [non-astrocytoma]; neuroradiologist assessment: negative). **e** T2-weighted image, **f** FLAIR image demonstrating persistent hyperintensity without central signal suppression, **g** T2-FLAIR digital subtraction image showing minimal signal intensity difference between sequences, **h** three-dimensional volume rendering depicting the segmented tumor volume (green overlay). Bottom row (i–l): IDH-mutant, 1p/19q non-codeleted, WHO Grade 2 diffuse astrocytoma (T2-FLAIR mismatch sign positive; radiomics model prediction: positive [astrocytoma]; neuroradiologist assessment: negative). This discordant case demonstrates a tumor where the radiomics model correctly identified the astrocytoma molecular subtype, while the neuroradiologist visual assessment did not identify the T2-FLAIR mismatch sign due to incomplete signal suppression on FLAIR. **i** T2-weighted image showing tumor hyperintensity, **j** FLAIR image demonstrating partial signal suppression that did not meet strict visual mismatch criteria, **k** T2-FLAIR digital subtraction image revealing quantifiable signal intensity differences captured by the radiomics model, **l** three-dimensional volume rendering depicting the segmented tumor volume (green overlay)

Figure 5 illustrates representative cases demonstrating the T2-FLAIR digital subtraction imaging process and three-dimensional tumor segmentation. The first case (top row, a–d) shows an IDH-mutant astrocytoma with the characteristic T2-FLAIR mismatch pattern, where a prominent signal difference is visible on the subtraction image; this case was correctly classified by both the radiomics model and the conventional neuroradiologist assessment. The second case (middle row, e–h) shows an IDH-mutant, 1p/19q co-deleted oligodendroglioma with persistent signal on both T2-weighted and FLAIR sequences without significant mismatch, resulting in minimal signal difference on the subtraction image; this case was also correctly classified by both methods. The third case (bottom row, i–l) demonstrates a clinically important discordant scenario: an IDH-mutant astrocytoma where the radiomics model correctly predicted the astrocytoma molecular subtype (positive), but the neuroradiologist visual assessment was negative because the partial signal suppression on FLAIR did not meet the strict visual criteria for the T2-FLAIR mismatch sign. This case illustrates the added value of the quantitative radiomics approach in detecting subtle mismatch patterns below the threshold of visual perception.

## Discussion

In this multi-center study, we developed and externally validated a radiomics-based machine learning model using T2-FLAIR digital subtraction images for differentiating IDH-mutant, 1p/19q non-codeleted astrocytomas from other non-enhancing LGG subtypes, including oligodendrogliomas and IDH-wildtype diffuse gliomas. The model achieved an AUC of 0.879 in the training cohort and 0.849 in the independent external test set, and statistically outperformed conventional neuroradiologist visual assessment of the T2-FLAIR mismatch sign (DeLong test: *p* = 0.003 and *p* = 0.038, respectively).

The clinical importance of accurately differentiating these molecular subtypes preoperatively cannot be overstated. IDH-mutant astrocytomas and IDH-mutant, 1p/19q co-deleted oligodendrogliomas, despite both being classified as lower-grade IDH-mutant gliomas, exhibit distinct biological behaviors and respond differently to treatment. Oligodendrogliomas demonstrate a particularly favorable response to PCV chemotherapy and carry a significantly better overall prognosis, with median survival exceeding 14 years compared to approximately 8–10 years for astrocytomas [4, 5]. Furthermore, surgical extent requirements and adjuvant treatment strategies differ: maximal safe resection followed by temozolomide-based chemotherapy is the standard for astrocytomas, whereas oligodendrogliomas may benefit more from PCV-based regimens [2]. Thus, reliable preoperative molecular subtype prediction has direct implications for treatment planning, the informed consent process, and patient counseling.

The conventional T2-FLAIR mismatch sign, as originally described by Patel et al. [6] and validated in subsequent studies [7–11, 20], exhibits high specificity (often approaching 100%) but limited sensitivity (22–51%) for identifying IDH-mutant, 1p/19q non-codeleted astrocytomas among LGGs. Our findings are consistent with these reports: the neuroradiologist assessment achieved specificities of 97.8% and 100.0% but sensitivities of only 56.1% and 40.9% in the training and external test cohorts, respectively. The radiomics model substantially improved sensitivity (to 71.2% and 86.4%, respectively) at the expense of a modest reduction in specificity (to 85.4% and 75.0%). This trade-off is clinically meaningful because the conventional assessment’s primary limitation—missing a substantial proportion of IDH-mutant astrocytomas—has direct consequences for treatment planning. The decreased specificity of the radiomics model relative to conventional assessment is an expected consequence of this sensitivity-specificity trade-off and reflects the model’s ability to identify subtler imaging patterns not captured by the strict visual criteria of the T2-FLAIR mismatch sign. Importantly, the radiomics model’s specificity (85.4% and 75.0%) remains clinically acceptable for a screening or decision-support tool, while the improved sensitivity may help identify astrocytoma cases that would otherwise be missed by conventional assessment alone.

The use of T2-FLAIR digital subtraction images as the basis for radiomics analysis represents a key methodological aspect of this study. By computing voxel-wise differences between registered T2 and FLAIR images, we directly quantified the imaging characteristics related to the T2-FLAIR mismatch phenomenon. This approach provides a more objective and comprehensive assessment compared to qualitative visual evaluation, capturing subtle variations in signal intensity relationships that may be difficult to perceive visually. The representative cases in Fig. 1 demonstrate how the subtraction images accentuate the differential signal characteristics between molecular subtypes, including a discordant case where the radiomics model correctly identified an astrocytoma despite a negative neuroradiologist visual assessment, underscoring the clinical value of this quantitative approach.

Our consensus feature selection approach, combining LASSO, mRMR, and Boruta methods, effectively reduced the dimensionality from 1,130 features to a parsimonious set of 10 highly discriminative features. This conservative feature selection strategy was crucial for preventing overfitting, as evidenced by the modest difference (0.030) between training and external test AUC values. The selected features predominantly represented first-order statistics (skewness, kurtosis) and texture features (GLCM correlation, sum squares), which capture signal intensity characteristics and spatial heterogeneity patterns on the subtraction images that differ between molecular subtypes.

The SHAP analysis provided insights into the features driving model predictions. First-order skewness emerged as the most influential feature, reflecting asymmetric signal intensity distributions on the subtraction images. However, it is important to acknowledge that radiomics features are statistical descriptors of image properties rather than direct biological markers [15, 16]. While the imaging patterns captured by these features likely reflect underlying differences in tumor composition (such as the presence of protoplasmic astrocytes with microcystic changes in astrocytomas), a direct causal link between specific radiomics features and tumor biology should be interpreted with caution. The prominence of wavelet-derived features suggests that multi-scale texture analysis captures relevant information that contributes to molecular subtype differentiation.

Several previous studies have explored radiomics and machine learning approaches for glioma molecular classification [18, 20, 22–25]. However, our approach differs in its specific use of T2-FLAIR digital subtraction imaging as the input for radiomics extraction. Choi et al. [26] demonstrated the value of deep learning combined with radiomics for predicting IDH mutation status, while Zhou et al. [27] developed a radiomics signature for 1p/19q codeletion prediction. Recent meta-analyses have highlighted the potential of machine learning-based approaches for glioma molecular classification, while also emphasizing the critical importance of external validation and standardized methodology [18, 19]. Our work complements these efforts by providing an automated method specifically leveraging the T2-FLAIR signal intensity relationship through digital subtraction.

The excellent inter-reader agreement (ICC = 0.89) for conventional T2-FLAIR mismatch assessment in our study is consistent with previous reports [14] and confirms the reliability of this visual assessment among experienced readers. However, the limited sensitivity of the mismatch sign (22–51% in published studies [6–11]) is fundamentally attributable to its strict binary visual definition rather than to reader experience. The mismatch sign requires complete or near-complete homogeneous signal suppression on FLAIR—a strict morphological criterion that inherently excludes partial or subtle mismatch patterns. We hypothesize that even highly experienced neuroradiologists with extensive subspecialty training (> 15–20 years) would demonstrate similar sensitivity limitations, as this constraint is inherent to the binary nature of the visual criterion rather than to the assessor’s perceptual ability. The radiomics model’s improved sensitivity reflects its capacity to quantify continuous, graded signal intensity differences on the digital subtraction images, capturing subtle variations that fall below the threshold of any categorical visual assessment regardless of assessor experience.

We did not investigate a combined model integrating radiomics features with conventional radiologist assessment in this study, as our primary objective was to evaluate an automated radiomics approach that could function independently of subjective visual assessment. Such a combined approach could potentially leverage the complementary strengths of both methods—the high specificity of conventional assessment and the improved sensitivity of radiomics—and merits investigation in future studies.

Several methodological considerations warrant discussion. First, we used the TCGA-LGG dataset as an external test set rather than as an additional training source, to obtain an unbiased estimate of model performance on completely independent data from different institutions and imaging protocols. While the TCGA-LGG cohort was relatively small (*n* = 38), it provided an important assessment of generalizability. Second, the class distribution shows a higher proportion of non-astrocytoma cases (57.4%) than astrocytomas (42.6%) in the training cohort, which differs from the expected higher prevalence of astrocytomas in unselected clinical populations. This may reflect selection characteristics of the EGD dataset and should be considered when interpreting the model’s predictive values. The use of balanced accuracy optimization during training and reporting of both sensitivity and specificity helps to mitigate the impact of this class distribution on performance assessment.

## Limitations

This study has several limitations. First, the retrospective design may introduce selection bias, and prospective validation is warranted. Second, imaging protocols varied across institutions and scanner vendors, although our standardized preprocessing pipeline (including N4 bias field correction, rigid registration, skull stripping, and intensity normalization) aimed to minimize these effects. Third, the external test cohort was relatively small (*n* = 38), and although it provided a preliminary assessment of generalizability, further validation in larger, prospective, multi-center cohorts is needed before clinical implementation. Fourth, we focused specifically on non-enhancing gliomas; the applicability of our model to contrast-enhancing tumors requires separate investigation. Fifth, the IDH-wildtype subgroup was small (*n* = 13 total), and the model’s performance for this specific molecular subtype cannot be reliably assessed independently. Sixth, the molecular subtype classification was binary (astrocytoma vs. other), which does not capture the full spectrum of glioma molecular heterogeneity. Finally, the radiomics features were extracted using a semi-automatic segmentation approach; fully automated segmentation pipelines may further improve the clinical practicality and reproducibility of this method.

## Clinical implications

The proposed radiomics-based model offers several potential clinical applications. First, it may serve as a quantitative screening tool to identify patients likely to have IDH-mutant astrocytoma, enabling focused radiological review and more targeted preoperative molecular workup. Second, the automated and reproducible nature of the radiomics analysis reduces inter-observer variability inherent to subjective visual assessment and enables consistent assessment quality across different clinical settings. Third, the model could be particularly valuable in centers with limited subspecialty neuroradiology expertise, where assessment of the T2-FLAIR mismatch sign may be less reliable. Fourth, the improved sensitivity of the radiomics approach compared to the conventional mismatch sign may help identify astrocytoma cases in the presurgical setting, potentially informing surgical strategy and adjuvant treatment planning. The clinical workflow would involve the following steps after standard MRI acquisition: (1) automated preprocessing of T2 and FLAIR sequences, (2) generation of the digital subtraction image, (3) tumor segmentation (currently semi-automatic, with potential for full automation using deep learning-based segmentation), (4) radiomics feature extraction, and (5) model-based prediction output. Future prospective studies should evaluate the clinical impact of this model on treatment decisions and patient outcomes in real-world settings.

## Conclusion

We developed and externally validated a radiomics-based machine learning model using T2-FLAIR digital subtraction images for differentiating IDH-mutant, 1p/19q non-codeleted astrocytomas from other non-enhancing LGG subtypes. The model achieved an AUC of 0.879 in the training cohort and 0.849 in the external test set (DeLong test: *p* = 0.003 and *p* = 0.038 vs. conventional assessment, respectively), with adequate generalizability across multi-center data. The primary advantage over conventional T2-FLAIR mismatch assessment was substantially improved sensitivity while maintaining acceptable specificity. This automated, quantitative approach may serve as a complementary tool for non-invasive molecular characterization of diffuse gliomas, particularly in identifying IDH-mutant astrocytomas that lack the conventional T2-FLAIR mismatch sign. 

## Data Availability

The TCGA-LGG dataset used in this study is publicly available at The Cancer Imaging Archive (https://www.cancerimagingarchive.net). The Erasmus Glioma Database (EGD) is available through the Erasmus MC Brain Tumor Center upon reasonable request. The radiomics feature extraction code and trained model will be made available upon reasonable request to the corresponding author.
